# Assessment of success of the Ponseti method of clubfoot management in sub-Saharan Africa: a systematic review

**DOI:** 10.1186/s12891-017-1814-8

**Published:** 2017-11-15

**Authors:** Tracey Smythe, Debra Mudariki, Hannah Kuper, Christopher Lavy, Allen Foster

**Affiliations:** 1 International Centre for Evidence in Disability, London School of Hygiene & Tropical Medicine, London, UK; 2 University of Witwatersrand, Johannesburg, South Africa; 3 Nuffield Department of Orthopaedics Rheumatology and Musculoskeletal Science, University of Oxford, Headington, UK

**Keywords:** Clubfoot, Congenital talipes equinovarus, Ponseti, Outcome, Evaluation, Treatment, Success, Africa, Sub-Sahara

## Abstract

**Background:**

Clubfoot is one of the most common congenital deformities affecting mobility. It leads to pain and disability if untreated. The Ponseti method is widely used for the correction of clubfoot. There is variation in how the result of clubfoot management is measured and reported. This review aims to determine and evaluate how success with the Ponseti method is reported in sub-Saharan Africa.

**Methods:**

Five databases were examined in August 2017 for studies that met the inclusion criteria of: (1) evaluation of the effect of clubfoot management; (2) use of the Ponseti method; (3) original study undertaken in sub-Saharan Africa; (4) published between 2000 and 2017. We used the PRISMA statement to report the scope of studies. The included studies were categorised according to a hierarchy of study methodologies and a 27-item quality measure identified methodological strengths and weaknesses. The definition of success was based on the primary outcome reported.

**Results:**

Seventy-seven articles were identified by the search. Twenty-two articles met the inclusion criteria, of which 14 (64%) reported a primary outcome. Outcomes were predominantly reported though case series and the quality of evidence was low. Clinical assessment was the most commonly reported outcome measure and few studies reported long-term outcome. The literature available to assess success of clubfoot management is characterised by a lack of standardisation of outcomes, with different measures reporting success in 68% to 98% of cases.

**Conclusion:**

We found variation in the criteria used to define success resulting in a wide range of results. There is need for an agreed definition of good outcome (successful management) following both the correction and the bracing phases of the Ponseti method to establish standards to monitor and evaluate service delivery.

**Electronic supplementary material:**

The online version of this article (10.1186/s12891-017-1814-8) contains supplementary material, which is available to authorized users.

## Background

Clubfoot, or congenital talipes equinovarus (CTEV), is one of the most common congenital musculoskeletal deformities. Within the Africa region, clubfoot birth prevalence is estimated as 1.11 (95%CI 0.96–1.26) per 1000 live births [1]. Untreated clubfoot results in pain, physical impairment and can ultimately cause disability [2]. The Ponseti method is widely used for the management of clubfoot [3]. It consists of two distinct phases, the correction phase and the maintenance phase [4]. The correction phase involves precise manipulation of the foot around the talus to correct the cavus, adductus and varus of the deformity. The manipulation position is held in a long leg plaster of paris cast and the cast is typically changed weekly. A percutaneous tenotomy of the Achilles tendon is usually performed to correct the residual equinus. The maintenance phase involves the use of a foot abduction brace (FAB) for 23 h a day for three months, followed by nightly use until four to five years of age [5].

Many classification systems have been proposed to assess the severity of the clubfoot deformity and to measure the impact of treatment [6]. Ponseti and Smoley [4] based their classification on clinical assessment of ankle dorsiflexion, heel varus, forefoot supination and tibial torsion after treatment. Feet were classified as good, acceptable or poor. Harrold and Walker [7] considered the extent of deformity correction. The Pirani score [8] and the Dimeglio score [9] are two of the most widely used classification systems for clubfoot deformity [10]. The Pirani score is from 0 to 6 where zero is a normal foot and six is the most severe deformity. It is reliable when used by non-specialist health workers [11]. The Dimeglio score has a maximum of 20 points and the deformity is graded as benign, moderate, severe or very severe.

Tools that have been developed to assess function include: assessment of patient satisfaction and pain, gait, heel position and range of motion [12, 13]; a questionnaire designed to measure overall satisfaction, foot appearance, pain and physical limitations [14]; and a detailed assessment of movement quality that requires mobility testing with a goniometer and muscle testing [15], but does not include parent reported outcomes.

There is a need for a standardised approach to report clubfoot treatment outcomes [16–18]. To address this gap, this review aims to investigate the literature and to determine and evaluate how success with the Ponseti method is reported in sub-Saharan Africa.

## Methods

### Search strategy

A systematic literature search was conducted in August 2017 for peer-reviewed articles presenting original research findings on the effect of treatment of clubfoot in children in sub-Saharan Africa. Studies were limited to outcomes of the Ponseti method as this technique is widely accepted as best practice [18]. There was no language restriction. Results are presented according to the PRISMA guidelines [19].

Excerpta Medica Database (EMBASE), Global Health, Medline, Africa Wide Information and African Journals Online were examined for studies meeting the following inclusion criteria: [1] evaluation of the effect of clubfoot management, [2] use of the Ponseti method, [3] original study undertaken in sub-Saharan Africa, and [4] published between 1st January 2000 and 1st August 2017. Concepts were expanded to include related terms and synonyms. A study was excluded if there was no evaluation of treatment, however there was no restriction on type of study to allow a quality assessment review. There was no limitation on age of children and the search was restricted by date (2000–2017) to capture current best practice. Full search terms are presented in Table 1 and the search terms for the country names are outlined in detail in Additional file 1.

**Table 1:** Search terms for treatment of clubfoot with the Ponseti method in sub-Saharan Africa

1	clubf??t or club-f??t or (club ADJ1 foot) or (talipes ADJ1 equinovarus) or (talipes ADJ1 equino-varus) or (congenital ADJ1 talipes ADJ1 equinovarus) or (congenital ADJ1 talipes ADJ1 equino-varus) or CTEV
2	Ponseti
3	Country name in sub-Saharan Africa^a^
4	1 AND 2 AND 3

^a^Outlined in detail in Additional file 1

All titles and abstracts were screened independently by two authors (TS and DM). The full paper was reviewed if selected by either author or if the abstract was absent. In addition, the reference lists of the included articles were screened. Consensus was reached through discussion where there was disagreement on eligibility.

### Data extraction

A pilot-tested spread-sheet was used for data extraction from articles that met the inclusion criteria. All characteristics recorded by one author (TS) were reviewed for accuracy by another author (DM). Data extracted included authors, year of publication, type of study, sample size, age of participants, duration of follow up and reported measurement of treatment outcome. Two authors [20, 21] were contacted to provide missing information. Where other forms of treatment were detailed or where a paper included a country outside of sub-Saharan Africa, only data regarding the Ponseti method and from the sub-Saharan African country were extracted.

### Assessment of study quality

Full articles that met the eligibility criteria were categorised according to a hierarchy of study methodologies [22] developed to assess intervention strategies used with children with developmental disabilities. Quality of evidence was ranked as:

I. Systematic review of randomised controlled trials (RCTs); RCT with *N* > 100
II. RCT with *N* < 100; Systematic review of cohort studies
III. Cohort studies with concurrent control group; Systematic reviews of case control studies
IV. Case series; Cohort study without concurrent control group; Case-control study
V. Expert opinion; Case study or report; Anecdotal Evidence.

In addition to the levels of evidence, we used a quality measure proposed by Downs and Black [23] to identify methodological strengths and weaknesses of the included studies as there was no limitation on type of study. The quality index is a 27-item checklist designed for use with both observational studies and randomised controlled trials. The index is comprised of five subscales: reporting (ten questions), external validity (three questions), internal validity (bias and confounding) (13 questions), and power (one question). Items are checked as ‘yes’, ‘partially’, ‘no’ or ‘unable to determine’ depending on the subscale and higher scores indicating higher quality. The maximum score is 32.

### Data analysis

The definition of success was determined by the primary outcome reported in the studies or if explicitly stated. There were no studies that were sufficiently homogenous in terms of participants and outcomes to include in a meta-analysis and data were not combined due to methodological and clinical heterogeneity. An integrative review method [15] that included problem identification, data presentation and analysis was used to incorporate results. Summary statistics for the quality measure were calculated and include the mean and range (minimum and maximum).

## Results

### Search results

**Fig. 1:**
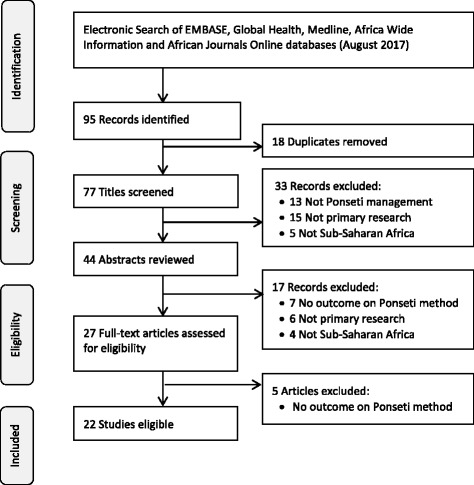
Search Strategy with PRISMA flow diagram

A total of seventy-seven articles were identified. Twenty-two studies met the inclusion criteria. The search strategy and reasons for excluding articles are presented in Fig. 1.

### Study characteristics

Characteristics of the eligible studies are presented in Table 2 and include children from one day old [21] to 10 years [24].

**Table 2:** Characteristics of studies that report outcomes of the Ponseti method in sub-Saharan Africa^a^

Primary Author Year Country	Number of children and (feet) treated	Age Range	Type of study (Level of Evidence)	Comparator Group	Duration of Follow up
Ibraheem 2017 [21], Nigeria	23 (14)	<3 months	Randomised controlled trial (II)	Children managed by accelerated Ponseti treatment	32–77 days.
Malagelada 2016 [32], South Africa	65 (91)	4–63 months	Cross sectional survey (IV)	Cases in a UK urban clinic	Not applicable
Smythe 2016 [35], Zimbabwe	173 (268)	17 days – 5 years 7 months	Case series, retrospective (IV)	Pre-treatment status of cases	10.2 weeks (9.5–10.9)
Boakye 2016 [38], Ghana	271 (430)	<6 months	Case series, Retrospective (IV)	Pre-treatment status of cases	Not reported
Adegbehingbe 2015 [39], Nigeria	4931 (7745)	Not reported	Case series (IV)	Pre-treatment status of cases	Not reported
Adewole 2014 [33], Nigeria	106 (158)	7 days – 4 years	Case series, prospective (IV)	Pre-treatment status of cases	Mean: 3 years (range 2–4)
Ayana 2014 [24], Ethiopia	22 (32)	2–10 years	Case series, prospective (IV)	Pre-treatment status of cases	Not reported
Kouamo 2014 [40], Togo	24 (41)	17 days - 7 years	Case series, prospective (IV)	Pre-treatment status of cases	Not applicable
Mang’oli 2014, Kenya	223 (361)	Mean 23 months	Cross sectional survey (IV)	Status of cases at previous appointment	One year
Kaseke 2013 [41], Zimbabwe	14 (20)	Mean 7.43 weeks	Non randomised, prospective (III)	Children managed with Kite technique	6 weeks
Adegbehingbe 2012 [42], Nigeria	493 (749)	Not reported	Case series, prospective (V)	Pre-treatment status of cases	Not reported
Cashman 2012 [20], Malawi	>2000	Not reported	Case series (IV)	No comparator	Not reported
Pirani 2012 [43], Uganda	370	Majority under 14 weeks	Case series, prospective (IV)	Pre-treatment status of cases	Not reported
Harnett 2011 [44], Malawi	21 (32)	<2 months	Randomised controlled trial (II)	Children managed by accelerated Ponseti treatment	Mean 258 days (70 to 348)
Adegbehingbe 2010 [25], Nigeria	55 (80)	<18 years	Randomised controlled trial (II)	Children treated by surgery	3–36 months post last cast
Radler 2010 [45], Mali	52	< 1 year	Case series (IV)	Pre-treatment status of cases	Not reported
Firth 2009 [30], South Africa	70 (106)	1 day – 40 months	Case series, retrospective (IV)	Pre-treatment status of cases	Mean: 2 years 5 months
Biruk 2007 [26], Ethiopia	55 (82)	< 6 months	Case series, prospective (IV)	Children in different age category	Not reported
Lavy 2007 [28], Malawi	307 (482)	<12 months	Case series, retrospective (IV)	Pre-treatment status of cases	Not reported
Khan 2005 [27], South Africa	(61)	Not reported	Case series (IV)	Pre-treatment status of cases	Not reported
Tindall 2005 [29], Malawi	75 (100)	Under 4 years	Case series, prospective (IV)	Pre-treatment status of cases	5 ft followed for 12-18 months
Mkandawire 2003 [36], Malawi	54	Under 2 years	Case series, Prospective (IV)	Pre-treatment status of cases	12 months

^a^Ordered by year of publication

The quality of evidence that reported outcomes of the Ponseti method in sub-Saharan Africa was low. Studies were included from ten countries in sub-Saharan Africa; studies undertaken in Nigeria and Malawi contributed five papers each. There were three RCTs, all with small sample sizes of less than 100 children. The majority of studies were classed as level IV [22] due to their observational nature.

### Definition of success – Primary outcome

All authors described a form of clinical assessment to assess outcome of treatment. Only 14 studies (64%) gave a clear definition of success. The Pirani score was defined as the primary outcome measure to assess the deformity correction in 14 studies. Change in the mean Dimeglio score was evaluated in one study [25] and frequency of initial severity was reported with the Harrold-Walker classification in two studies [26, 27]. Other definitions of primary outcome included: the number of days in casts [21], number of patients treated without extensive surgery [25], a plantigrade foot [24, 28, 29], no residual deformity [30], deformity status compared to previous visits [31] and parent reported outcomes on impact of treatment [32]. Limited definition terms included “complete correction” [26] and “satisfactory outcome” [25]. The approach to reporting severity scores varied (Table 3).

**Table 3:** Reported Primary Outcome using the Ponseti method in sub-Saharan Africa

Primary Author Year Country*	Clubfoot severity assessment	Reported Success Measure	Recurrence / relapse	Additional surgical intervention
Ibraheem 2017 [21], Nigeria	Pirani score	Number of days in casts, number of casts applied	Not reported	Not reported
Malagelada 2016 [32], South Africa	Pirani score	Parent reported outcomes	12% (8 children)	Not reported
Smythe 2016 [35], Zimbabwe	Pirani Score	85% feet; Pirani score < 1	Not reported	Not reported
Boakye 2016 [38], Ghana	Pirani Score	Number of casts to correction. Correction not defined.	Not reported	Not reported
Adegbehingbe 2015 [39], Nigeria	Not reported	89.7% (4426 patients) satisfactory outcome. Criteria for satisfactory outcome not defined.	4% (253 feet, 194 patients)	3%
Adewole 2014 [33], Nigeria	Pirani score and photograph	100%; based on clinical judgement, Plantigrade functional foot	5.16% (8 feet)	6 feet
Ayana 2014 [24], Ethiopia	Pirani score	28/41 good results Good = correction of all deformities. 97.8% achieved score of <3	2 patients, 4 feet	8 children/ (11 feet)
Kouamo 2014 [40], Togo	Not reported	94% (179/190) compliant with brace wear 93.5% no visible discomfort	12.2% (5 cases)	Not reported
Mang’oli 2014 , Kenya	Pirani score	Initial correction: 96.2% (152 feet) Initial correction not defined.	Not reported	Not reported
Kaseke 2013 [41], Zimbabwe	Pirani score	Rate of correction: Pirani score at 3 weeks and 6 weeks	Not reported	Not reported
Adegbehingbe 2012 [42], Nigeria	Pirani Score	89.7% treated successfully. Criteria for success not defined.	Not reported	3.2% (16 patients)
Cashman 2012 [20], Malawi	Not reported	30 children failed treatment (required more extensive surgery)	Not reported	30 children
Pirani 2012 [43], Uganda	Pirani Score	Mean score 5.4 falls to <2 by cast 6. Primary outcome not defined.	Not reported	Not reported
Harnett 2011 [44], Malawi	Pirani Score	Pirani score change. Median start Pirani: 5 (4 to 6). Median at tenotomy /end treatment: 0.5 (0.5 to 1) Median at 6 months: 0.5 (0 to 0.5)	No episodes of recurrence after 6 months	3 patients not corrected (7%) with Pirani >1
Adegbehingbe 2010 [25], Nigeria	Dimeglio classification	96.4% (53/55 children) = satisfactory (No recurrence) 3.6% (2/55) = fair (recurrence corrected with casts/FAB) Nil = poor (recurrence with repeat surgery)	2 had recurrence between 4 and 6 months	None
Radler 2010 [45], Mali	Not reported	77% (40 children): good or average. 23% (12 children): poor. Primary outcome not defined.	Not reported	Not reported
Firth 2009 [30], South Africa	Pirani score	61% fully corrected without residual deformity	23% (re-plaster 24 feet) 39% (41 feet mild recurrence)	7% (7 feet)
Biruk 2007 [26], Ethiopia	Harrold-Walker classification	76.8% (63 feet) No definition of complete correction.	Not reported	Not reported for Ponseti cohort
Lavy 2007 [28], Malawi	Pirani score	68% (327/482) Plantigrade or better	Not reported	12 children referred for surgery
Khan 2005 [27], South Africa	Harrold-Walker classification	6 failures from 61 feet. Criteria for success not defined.	Not reported	Not reported
Tindall 2005 [29], Malawi	Pirani score	98% plantigrade foot with Pirani score	Not reported	2%
Mkandawire 2003 [36], Malawi	Pirani score	Correction of deformity. Success of correction defined as fitting brace. Mean Pirani score decreased from 3.6–0.86	4 children with untreated clubfoot, 5 with complex and 7 with teratologic	Not reported

*Ordered by year of publication

### Process outcomes

There was wide variation in the measurement of process outcomes. The point in treatment when the number of casts was calculated was either before or after the final post tenotomy cast and was inconsistently described. Studies either reported frequency of tenotomy per child or per foot. Definition of relapse or recurrence of deformity differed in the included studies and technical details were only described in five studies (23%).

Six studies report on brace use [25, 28, 30–33] with the focus on non-compliance. Non-compliance was not well defined in the studies and varied from 2% to 44%.

One study assessed parent reported outcomes. The study aimed to determine the impact of the casting and bracing phases of the Ponseti method on the family. Each caregiver completed three questionnaires [32] in order to examine the level of impact that Ponseti treatment had on lives of caregivers and the coping strategies employed.

Reported process outcomes are presented in Table 4.

**Table 4:** Outcomes of the Ponseti Method reported in sub-Saharan Africa^a^

Primary Author (Year) Country	Process Outcomes						
	Average number of casts	Duration of casts	Percutaneous Achilles Tenotomy	Receipt of braces	Brace compliance	Loss to follow up	Complications
Ibraheem (2017 Nigeria	5.43	52 days (35–77)	1 child did not have tenotomy, not reported case or control	100%	Not reported	Nil	Reported no complications with swelling
Malagelada (2016) South Africa	8.7 (range 1–24)	Not reported	89% (58 children)	100% due to inclusion criteria	2% (1 child) non-compliant	Not applicable	Defined as relapse and non-compliance: 9 children
Smythe (2016) Zimbabwe	7.27 (6.7–7.9)	10.2 (9.5–10.9) weeks included tenotomy	78.9% (127/161 children)	Not reported	Not reported	8.9% (17 children)	Not reported
Boakye (2016) Ghana	4.93	Not reported	77%	Not reported	Not reported	Excluded from analysis	Not reported
Adegbehingbe (2015) Nigeria	Not reported	Not reported	77% (5626 children)	Not reported	Not reported	Not reported	Not reported
Adweole (2014) Nigeria	4.6 (range 3–9)	Weekly cast change, tenotomy 3 weeks	26.6% (42 feet)	56.8% (60 patients)	No child with relapse wore braces	Not reported	9 feet: cast complications, blisters, ulcers, skin rash
Ayana (2014) Ethiopia	8 (range 6–10)	Casts changed every 2 weeks	63.6% (14 children, 21 feet)	100%; < 4 yrs. = FAB >4 yrs. = ankle foot orthosis	Not reported	1 patient	No major complications
Kouamo (2014) Togo	Not reported	Not reported	82.9% (34/41 feet)	Not reported	Not reported	Not reported	Not reported
Mangoli (2014) Kenya	Not reported	Not reported	Not reported	100% of interviewed parents	15% (33/223) non-compliant Mean use 18 months (6–23)	Not applicable	5% (11/223) skin lesion
Kaseke (2013) Zimbabwe	Not reported	Not reported	Not reported	Not reported	Not reported	6 feet not reported at 6 weeks	Not reported
Adegbehingbe (2012) Nigeria	Not reported	Not reported	Not reported	Not reported	Not reported	Not reported	Not reported
Cashman (2012) Malawi	Not reported	Not reported	>80%	Not reported	Not reported	107 children	Not reported
Pirani (2012) Uganda	Not reported	Majority corrected by 6th treatment’	Not reported	Not reported	Not reported	83% adherence rate to end of casting	Plaster burns in 19/1000
Harnett (2011) Malawi	Median 5 (4–7)	42 days (35–84) in plaster prior to tenotomy.	52% (11 children)	Given FAB to wear until 3 years old	Not reported	2 after plaster. 1 patient died	Not reported
Adegbehingbe (2010) Nigeria	≤ 6 (76.4%; range 2–6) >6 (23.6% range 7–10)	2.3–13.7 +/−1.7 weeks	5.5% (3 children)	Not reported	Noted as ‘generally good’	None, not explicitly mentioned	3.6% ugly scar, recurrence, blister, infection
Radler (2010) Mali	Not reported	Not reported	Not reported	Not reported	Not reported	Not reported	Not reported
Firth (2009) South Africa	6.5 (range 2–18)	Not reported	74% (78 feet)	Received FABs, % unspecified	16% (11 patients) non-compliant	Not reported	8% (9 feet) minor blistering from braces
Biruk (2007) Ethiopia	Maximum cast 17 times	Weekly cast change	Not reported	60%, average wait time 3-4 months	Not reported	Not reported	Not reported for Ponseti cohort
Lavy (2007) Malawi	Not reported	Not reported	37% had tenotomy	44% given FABs	44% (145/327 feet)	32% (155 feet)	307 adequate records
Khan (2005) South Africa	Not reported	Not reported	Not reported	Not reported	Not reported	Not reported	Not reported
Tindall (2005) Malawi	5.3	Mean treatment 9.1 weeks	41%	All	Not reported	Not reported	2 minor complications
Mkandawire (2003) Malawi	Weekly cast change	Mean treatment: 7.4 weeks for idiopathic, 7.1 weeks for complex	Not reported	Not reported	Not reported	32 patients (35%)	Not reported

^a^Ordered by year of publication

According to the quality assessment (Additional file 2 outlines the individual study results using the Downs and Black (1998) criteria), the mean quality score of the included studies was 14.8 (5–21).

### Reporting

Reporting was the highest scoring category of the quality assessment. All studies included a clear study hypothesis and aim and the majority (17/22) clearly described the characteristics of the patients and the intervention. However, while some distributions of principle confounders were partially described, few studies accounted for confounding in the study design or analysis. Loss to follow up was only reported in half of the studies. Few studies demonstrated a comprehensive attempt to measure adverse effects.

### External validity

Many children were recruited from University and tertiary hospitals or national centres and therefore external validity was limited as the interventions undertaken in a specialist centre are likely unrepresentative of the hospitals most of the source population would attend.

### Internal validity – Bias and confounding

Randomisation is not possible in cohort studies and in the studies where randomisation was used, it was not possible to determine if the intervention assignment was concealed from both parents and staff until recruitment was complete and irrevocable. Characteristics of losses of patient follow up were inconsistently taken into account and reported in seven (32%) studies. Statistical tests used to assess the main outcomes and why they were chosen were inconsistently described; for example, median, mean and maximum of the number of casts used to achieve correction are reported in different papers. Power calculations were only outlined in three studies.

## Discussion

This literature review comprises results from case series, prospective trials and cross-sectional surveys in sub-Saharan Africa. There were few comparative studies concerning the Ponseti method in the region and there were no agreed protocols for reporting the results and outcome of treatment. Due to ethical considerations, most trials investigating treatment of clubfoot are not randomised controlled trials (RCTs) but comparisons of treatments or a review of cohort outcomes. Potential sources of bias in observational studies are well documented [34] and whilst systematic reviews of health care interventions most often focus on RCTs, the inclusion of cohort studies in this review highlights the need for quality design and reporting of studies to increase the strength of evidence.

### Principal findings and considerations

A definition of a primary outcome (success) was described in 14 of the 22 studies. Successful outcome ranged from 68% to 98% of cases using different definitions in the 14 studies. There was no consensus on how to define a successful outcome of treatment. There was selective reporting of positive results with little detail given to treatment failure [35]. A range of process measures was included in the studies. The mean number of casts required ranged from 4.6 to 8.7 and is likely affected by the point at which the last cast was measured (pre- or post-tenotomy) and the unlimited age range of the review criteria. The studies used different criteria for relapse recognition and management. Two studies reported patient attrition over 30% [28, 36] however the length of follow-up in the majority of studies was short and few data were available on characteristics of children lost to follow up.

Acknowledging the limitations of the available reported papers, this review suggests that the Ponseti method appears to give successful correction of clubfoot during the correction phase when measured by the Pirani score, Dimeglio classification or simple clinical assessment. However, the lack of a consistent measure of success and insufficient follow up of cases restricts the conclusions that can be made about what happens during the bracing phase, be it success, recurrence or loss to follow-up.

### Main findings as related to other publications

The included studies report success in 68% to 98% of cases after the correction (casting) phase. In contrast, global success rates after the correction phase are cited as approximately 90% [18, 37]. Comprehensive tools to assess function (e.g. as described by Laaveg and Ponseti (12), the Roye tool [14], the Bangla tool [13] or the Clubfoot Assessment Protocol (CAP) [15]) are not reported in the studies from sub-Saharan Africa.

### Implications of findings

We found that the differences between study populations, methodology and the way that outcomes are described contribute to the variation in results reported for the Ponseti method in sub-Saharan Africa. Currently, different scores are used for the assessment of clubfoot severity. Standardisation is required to define successful outcome of clubfoot management so that risk factors for good and poor outcome can be determined and services can be monitored and evaluated.

The Pirani score was the most frequent clinical assessment used. It has been validated in younger children and demonstrates acceptable interrater reliability [8]. A short assessment time is required and it is easy to use, however to ensure consistency more guidance would be helpful on how to measure the individual components, as similarly provided by the diagrams and video produced to aid assessment with the Dimeglio score. The Pirani scoring system is the only assessment that has evidence for use by paramedics, and is in our opinion the easiest severity measure to use in young children before walking age.

### Methodologic issues

To our knowledge, this is the first systematic review of outcomes to measure success of the Ponseti method in sub-Saharan Africa. The observation of explicit methodology and lack of language restriction are strengths of this study. The literature available to assess success of clubfoot treatment is characterised by a lack of standardisation of outcomes. Studies routinely use the term “success rates” but do not define a successful outcome. Given that Ponseti management involves both correction and maintenance, the definition of success should always reflect both of these important endpoints and we encourage researchers to measure and report both. Bias in internal validity arose from studies where differences in follow up were regularly ignored, however compliance with the corrective phase of the intervention was generally reported as being good. Studies must include follow-up or acknowledge the limitations of selecting one part of the treatment process.

The potential for confounding in the reviewed studies to obscure true effects is significant as the majority are observational. Randomisation may be considered unethical in certain circumstances and well designed controlled trials may provide more opportunities to analyse different outcomes. Studies intended to address comparative effectiveness of management for clubfoot should use a careful control for covariates such as unilateral or bilateral clubfoot as disproportionate weighting is given to bilateral cases [17].

### Research gaps

Although a number of studies are available on initial treatment (correction phase) outcomes, very few studies are available on long term outcomes and follow up in the bracing phase, which are essential for measuring success of the entire Ponseti method.

No study compared different scoring systems. A study comparing multiple assessments in the same patient before and after treatment would be of value in assessing the equivalence or superiority of measurement techniques.

Studies need to control for the side of clubfoot and previous treatment, account for loss to follow up and adjust for confounding in methods or analysis in order to avoid the shortfalls of the current observational literature.

### Recommendations

Consensus is needed to standardise the reporting of outcomes and how success after Ponseti management is defined. For sub-Saharan Africa the definition needs to be appropriate for use by trained therapists who are managing children with clubfoot. This systematic review contributes to the knowledge about the importance of providing evidence to improve clubfoot services.

## Conclusions

The lack of good quality studies, variation in definition of success and limited follow-up of patients means the success rate of clubfoot treatment using the Ponseti method in sub-Saharan Africa is uncertain. There is need for an agreed definition of good outcome following both the correction and the bracing phase to monitor and evaluate service delivery and identify reasons for poor outcome. It is very important that children who complete the correction phase are followed through the bracing phase and results on success, recurrence and loss to follow up are reported. Studies are also required to document the correlation between clinical outcome, functional outcome and patient/family reported satisfaction.

## Additional files

Additional file 1: Expanded search terms for country name in sub-Saharan Africa. (DOCX 13 kb)

Additional file 2: Quality index assessment for included studies (studies 1–11 assessed on pages 1–3 and studies 12–22 assessed on pages 4–6). (DOCX 35 kb)

## Abbreviations

CTEV: Congenital Talipes Equinovarus
FAB: Foot Abduction Brace
PRISMA: Preferred Reporting Items for Systematic Reviews and Meta-Analyses
