# Supplementary material for: Assessment of success of the Ponseti method of clubfoot management in sub-Saharan Africa: a systematic review
Source: BMC Musculoskelet Disord. 2017 Nov 15;18:453. doi: 10.1186/s12891-017-1814-8 (PMC5688674; doi:10.1186/s12891-017-1814-8)
Supplement: Supplementary file 2 — Quality index assessment for included studies (studies 1–11 assessed on pages 1–3 and studies 12–22 assessed on pages 4–6). (DOCX 35 kb) [file 12891_2017_1814_MOESM2_ESM.docx]

**Additional file 2: Appendix 2. Quality Index assessment for included studies (studies 1-11 assessed on pages 1-3 and studies 12-22 assessed on pages 4-6)**

| Quality Index (Downs & Black, 1998, pp. 382-383)  **(papers 1- 11)** | Ibraheem 2017, Nigeria | Malagelada 2016, South Africa | Smythe 2016, Zimbabwe | Boakye 2016, Ghana | Adegbehingbe 2015, Nigeria | Adewole 2014, Nigeria | Ayana 2014, Ethiopia | Kouamo 2014, Togo | Mang'oli 2014, Kenya | Kaseke 2013, Zimbabwe | Adegbehingbe 2012, Nigeria |
| --- | --- | --- | --- | --- | --- | --- | --- | --- | --- | --- | --- |
| **Reporting: Were the following clearly described? (Y/N)** |  |  |  |  |  |  |  |  |  |  |  |
| 1. Study hypothesis/aim/objective | 1 | 1 | 1 | 1 | 1 | 1 | 1 | 1 | 1 | 1 | 1 |
| 2. Main outcomes | 1 | 1 | 1 | 0 | 0 | 1 | 1 | 1 | 0 | 1 | 0 |
| 3. Characteristics of the participants | 1 | 1 | 1 | 1 | 0 | 1 | 1 | 1 | 1 | 0 | 1 |
| 4. Interventions of interest | 1 | 1 | 1 | 1 | 0 | 1 | 1 | 1 | 1 | 0 | 0 |
| 5. Distributions of principal confounders in each group | 1 | 1 | 1 | 1 | 0 | 1 | 0 | 0 | 0 | 0 | 0 |
| 6. Main findings | 1 | 1 | 1 | 1 | 1 | 1 | 1 | 1 | 1 | 1 | 1 |
| 7. Estimates of random variability for main outcomes | 1 | 1 | 1 | 1 | 0 | 1 | 1 | 0 | 0 | 1 | 0 |
| 8. All the important adverse events that may be a consequence of intervention are reported | 0 | 0 | 0 | 0 | 0 | 1 | 1 | 0 | 0 | 0 | 0 |
| 9. Characteristics of patients lost to follow-up | 1 | 1 | 1 | 0 | 0 | 0 | 1 | 0 | 1 | 0 | 0 |
| 10. Actual probability values for main outcomes | 1 | 1 | 1 | 1 | 0 | 0 | 0 | 0 | 0 | 1 | 1 |
| **External validity (Y/N/unable to determine)** |  |  |  |  |  |  |  |  |  |  |  |
| 11. Were subjects who were asked to participate representative of the entire population from which they were recruited? | 1 | 0 | 1 | 1 | 1 | 1 | 1 | 0 | 0 | 1 | 1 |
| 12. Were subjects who were prepared to participate representative of the entire population from which they were recruited? | 1 | 0 | 1 | 1 | 1 | 1 | 1 | 0 | 0 | 1 | 1 |
| 13. Were the staff, places, and facilities representative of the treatment the majority of subjects received? | 0 | 0 | 0 | 0 | 1 | 0 | 0 | 0 | 1 | 0 | 0 |
| **Internal validity – bias (Y/N/unable to determine)** |  |  |  |  |  |  |  |  |  |  |  |
| 14. Was an attempt made to blind subjects to the intervention they received? | 0 | 1 | 0 | 0 | 0 | 0 | 0 | 0 | 0 | 0 | 0 |
| 15. Was an attempt made to blind those measuring main outcomes of the intervention? | 0 | 0 | 0 | 0 | 0 | 0 | 0 | 0 | 0 | 0 | 0 |
| 16. If any of the results of the study were based on “data dredging” was this made clear? | 1 | 1 | 1 | 1 | 0 | 1 | 1 | 1 | 1 | 1 | 1 |
| 17. In trials and cohort studies, do analyses adjust for different lengths of follow-up? Or, in case-control studies, is the period between intervention and outcome the same for cases and controls? | 1 | 1 | 1 | 1 | 0 | 1 | 1 | 1 | 1 | 1 | 1 |
| 18. Were appropriate statistical tests used to assess the main outcomes? | 1 | 1 | 1 | 1 | 0 | 1 | 1 | 1 | 1 | 1 | 1 |
| 19. Was compliance with the intervention reliable? | 1 | 1 | 1 | 1 | 0 | 1 | 1 | 1 | 1 | 0 | 1 |
| 20. Were main outcome measures reliable and valid? | 1 | 1 | 1 | 1 | 0 | 1 | 1 | 1 | 1 | 0 | 1 |
| **Internal validity – confounding (selection bias) (Y/N/unable to determine)** |  |  |  |  |  |  |  |  |  |  |  |
| 21. For trials and cohort studies, were patients in different intervention groups? For case-control studies, were cases and controls recruited from the same population? | 1 | 1 | 1 | 1 | 1 | 1 | 1 | 1 | 1 | 1 | 1 |
| 22. Were study subjects in different intervention groups (trials and cohort studies) or were the cases and controls (case-control studies) recruited over the same period of time? | 1 | 0 | 1 | 1 | 0 | 1 | 1 | 1 | 1 | 0 | 1 |
| 23. Were subjects randomized to intervention groups? | 1 | 0 | 0 | 0 | 0 | 0 | 0 | 0 | 0 | 0 | 0 |
| 24. Was the randomized intervention assignment concealed from both patients and staff until recruitment was complete and irrevocable? | 0 | 0 | 0 | 0 | 0 | 0 | 0 | 0 | 0 | 0 | 0 |
| 25. Was there adequate adjustment for confounding in the analyses from which main findings were drawn? | 0 | 0 | 1 | 0 | 0 | 0 | 0 | 0 | 0 | 0 | 0 |
| 26. Were losses of subjects to follow-up taken into account? | 1 | 1 | 1 | 1 | 0 | 0 | 1 | 0 | 1 | 0 | 0 |
| **Power** |  |  |  |  |  |  |  |  |  |  |  |
| 27. Did the study have sufficient power to detect a clinically important effect where the probability for a difference due to chance was less than 5%? | 0 | 0 | 0 | 0 | 0 | 0 | 0 | 0 | 0 | 1 | 0 |

| Quality Index (Downs & Black, 1998, pp. 382-383)  **(papers 12-22)** | Cashman 2012, Malawi | Pirani 2012, Uganda | Harnett 2011, Malawi | Adegbehingbe 2010, Nigeria | Radler 2010, Mali | Firth 2009, South Africa | Biruk 2007, Ethiopia | Lavy 2007, Malawi | Khan 2005, South Africa | Tindall 2005, Malawi | Mkandawire 2003, Malawi |
| --- | --- | --- | --- | --- | --- | --- | --- | --- | --- | --- | --- |
| **Reporting: Were the following clearly described? (Y/N)** |  |  |  |  |  |  |  |  |  |  |  |
| 1. Study hypothesis/aim/objective | 1 | 1 | 1 | 1 | 1 | 1 | 1 | 1 | 1 | 1 | 1 |
| 2. Main outcomes | 1 | 0 | 1 | 1 | 0 | 1 | 0 | 1 | 0 | 1 | 1 |
| 3. Characteristics of the participants | 0 | 1 | 1 | 1 | 0 | 1 | 1 | 1 | 0 | 1 | 1 |
| 4. Interventions of interest | 0 | 1 | 1 | 1 | 1 | 1 | 1 | 1 | 1 | 1 | 1 |
| 5. Distributions of principal confounders in each group | 0 | 1 | 1 | 1 | 0 | 1 | 0 | 1 | 0 | 1 | 1 |
| 6. Main findings | 1 | 1 | 1 | 1 | 1 | 1 | 1 | 1 | 0 | 1 | 1 |
| 7. Estimates of random variability for main outcomes | 0 | 0 | 1 | 1 | 0 | 1 | 1 | 1 | 0 | 1 | 1 |
| 8. All the important adverse events that may be a consequence of intervention are reported | 0 | 1 | 1 | 1 | 0 | 1 | 1 | 0 | 0 | 1 | 0 |
| 9. Characteristics of patients lost to follow-up | 0 | 0 | 1 | 0 | 0 | 0 | 0 | 0 | 0 | 1 | 0 |
| 10. Actual probability values for main outcomes | 0 | 0 | 1 | 1 | 0 | 1 | 0 | 0 | 0 | 0 | 0 |
| **External validity (Y/N/unable to determine)** |  |  |  |  |  |  |  |  |  |  |  |
| 11. Were subjects who were asked to participate representative of the entire population from which they were recruited? | 1 | 1 | 1 | 1 | 0 | 0 | 1 | 1 | 0 | 1 | 1 |
| 12. Were subjects who were prepared to participate representative of the entire population from which they were recruited? | 1 | 1 | 1 | 1 | 0 | 0 | 1 | 0 | 0 | 1 | 1 |
| 13. Were the staff, places, and facilities representative of the treatment the majority of subjects received? | 1 | 1 | 0 | 0 | 0 | 0 | 0 | 1 | 0 | 0 | 0 |
| **Internal validity – bias (Y/N/unable to determine)** |  |  |  |  |  |  |  |  |  |  |  |
| 14. Was an attempt made to blind subjects to the intervention they received? | 0 | 0 | 0 | 1 | 0 | 0 | 0 | 0 | 0 | 0 | 0 |
| 15. Was an attempt made to blind those measuring main outcomes of the intervention? | 0 | 0 | 1 | 1 | 0 | 0 | 0 | 0 | 0 | 0 | 0 |
| 16. If any of the results of the study were based on “data dredging” was this made clear? | 1 | 1 | 1 | 1 | 1 | 1 | 1 | 1 | 1 | 1 | 1 |
| 17. In trials and cohort studies, do analyses adjust for different lengths of follow-up? Or, in case-control studies, is the period between intervention and outcome the same for cases and controls? | 0 | 1 | 1 | 1 | 1 | 0 | 0 | 1 | 1 | 1 | 1 |
| 18. Were appropriate statistical tests used to assess the main outcomes? | 0 | 1 | 1 | 1 | 0 | 1 | 1 | 1 | 1 | 1 | 1 |
| 19. Was compliance with the intervention reliable? | 0 | 1 | 1 | 1 | 0 | 1 | 0 | 0 | 0 | 1 | 0 |
| 20. Were main outcome measures reliable and valid? | 0 | 1 | 1 | 1 | 0 | 1 | 0 | 1 | 0 | 1 | 1 |
| **Internal validity – confounding (selection bias) (Y/N/unable to determine)** |  |  |  |  |  |  |  |  |  |  |  |
| 21. For trials and cohort studies, were patients in different intervention groups? For case-control studies, were cases and controls recruited from the same population? | 1 | 1 | 1 | 1 | 1 | 1 | 1 | 1 | 0 | 1 | 1 |
| 22. Were study subjects in different intervention groups (trials and cohort studies) or were the cases and controls (case-control studies) recruited over the same period of time? | 1 | 1 | 1 | 1 | 1 | 1 | 1 | 1 | 0 | 1 | 1 |
| 23. Were subjects randomized to intervention groups? | 0 | 0 | 1 | 1 | 0 | 0 | 0 | 0 | 0 | 0 | 0 |
| 24. Was the randomized intervention assignment concealed from both patients and staff until recruitment was complete and irrevocable? | 0 | 0 | 0 | 0 | 0 | 0 | 0 | 0 | 0 | 0 | 0 |
| 25. Was there adequate adjustment for confounding in the analyses from which main findings were drawn? | 0 | 0 | 0 | 0 | 0 | 0 | 0 | 0 | 0 | 0 | 0 |
| 26. Were losses of subjects to follow-up taken into account? | 0 | 0 | 1 | 0 | 0 | 0 | 0 | 0 | 0 | 1 | 1 |
| **Power** |  |  |  |  |  |  |  |  |  |  |  |
| 27. Did the study have sufficient power to detect a clinically important effect where the probability for a difference due to chance was less than 5%? | 0 | 0 | 1 | 0 | 0 | 0 | 1 | 0 | 0 | 0 | 0 |
